# Shaping more resilient and just food systems: Lessons from the COVID-19 Pandemic

**DOI:** 10.1007/s13280-021-01532-y

**Published:** 2021-03-09

**Authors:** Angelina Sanderson Bellamy, Ella Furness, Poppy Nicol, Hannah Pitt, Alice Taherzadeh

**Affiliations:** 1 Sustainable Places Research Institute, Cardiff University, 33 Park Place, CF10 3BA Cardiff, UK; 2 School of Biosciences, Cardiff University, Cardiff, UK; 3 School of Geography and Planning, Cardiff University, Cardiff, UK; 4 School of Social Sciences, Cardiff University, Cardiff, UK

**Keywords:** COVID-19 pandemic, Food justice, Food systems, Resilience, UK

## Abstract

The COVID-19 pandemic has highlighted weaknesses in global food systems, as well as opening windows of opportunity for innovation and transformation. While the nature and extent of this crisis is rare, extreme climatic events will increase in magnitude and frequency, threatening similar societal impacts. It is therefore critical to identify mechanisms for developing food systems that are resilient to such impacts. We examine impacts of the crisis on UK food systems and how these further entrenched social inequalities. We present data on the experiences and actions of producers, consumers, and community organisers. The data were collected by adapting ongoing research to include surveys, interviews and online workshops focused on the pandemic. Actors’ responses to the pandemic foreshadow how enduring change to food systems can be achieved. We identify support required to enable these transformations and argue that it is vital that these opportunities are embedded in food justice principles which promote people-centred approaches to avoid exacerbating injustices prevalent pre-crisis. Learning from these experiences therefore provides insights for how to make food systems elsewhere more resilient and just.

## Introduction

Prior to the COVID-19 pandemic, global food systems were plagued by interlinked crises of obesity (Swinburn et al. 2019), malnutrition, poverty (FAO et al. 2019), climate change (IPCC 2019), ecosystem degradation, and biodiversity loss (Hayhow 2019; IPBES 2019). Links between intensive industrial food systems, ill-health, environmental damage, and injustice (Frison and Clément 2020) reveal an urgent need for sustainable, just food systems. Concentration of power and profits among global corporate elites (Levkoe and Sheedy 2019, p. 328) has resulted in ‘place-less’, homogeneous foodscapes disconnected from distinct territories and diverse socio-cultural practices. As indicated by the EAT Lancet Commission: “food systems have the potential to nurture human health and support environmental sustainability; however, they are currently threatening both” (Willett et al. 2019, p. 447). Many of the world’s most vulnerable people are worst affected by these interlocking crises.

Long-term stressors have eroded food systems’ resilience globally, making them vulnerable to shorter-term shocks like COVID-19. The pandemic highlights multiple vulnerabilities in food systems, including dominance of a small pool of retailers and producers, reliance on just-in-time supply chains, and dependence on imported food and labour (Garnett et al. 2020). While food systems have long failed to meet the needs of people equitably (Levkoe 2014; FAO 2019), the pandemic further highlights current injustices in food systems. In combination with widespread job losses, reduced household incomes and rising food prices (Naidu-Ghelani 2020), these pressures increase the severity of household food insecurity, exacerbating pre-existing poverty and inequalities (O’Connell et al. 2019). The upswell of support for Black Lives Matter during the pandemic highlights the need to address injustices including racism when tackling power asymmetries within current food systems. But in times of crisis urgency might override other concerns, meaning decision-making has the potential to further entrench injustice and fail to include marginalised voices (Maughan et al. 2020).

While the impacts of COVID-19 may be temporary, food systems will suffer other crises such as extreme climatic events which will likely increase in severity and frequency, leading to similar societal impacts around the world (IPCC 2019). It is therefore crucial to understand how the current global pandemic is affecting food systems and learn from responses—both positive and negative—so reactions to future crises can promote just and sustainable outcomes. Here we share evidence of COVID-19’s impact on UK food systems from the perspective of producers, consumers and community organisers, seeking insights to crisis impacts and responses in the context of food systems which are neither resilient nor just. These disruptions present windows of opportunity through which these actors are pushing for change in ways that foreshadow how enduring change could be achieved. We identify governmental support and policies required to enable transformations and argue that ‘building back better’ food systems must include social and environmental justice.

### Inequalities and COVID-19

The pandemic has intensified injustices experienced by those already vulnerable to the current shortcomings of the food system. In the UK, it is estimated that the number of people experiencing food insecurity quadrupled due to the COVID-19 pandemic. This has been attributed to lack of food in shops, economic impacts, and isolation (Loopstra 2020). The pandemic has exacerbated food insecurity for those already struggling, while creating new vulnerabilities through lost income or work (Loopstra 2020). During the first six weeks of lockdown in the UK 72% of parents eligible for free school meals were worried about getting enough vegetables, while Black, Asian and minority ethnic (BAME) households were found to experience greater than average food insecurity (Food Foundation 2020).

The pandemic also reveals interconnections between social inequalities, food insecurity and ill-health. Dietary related non-communicable diseases (NCDs) are a risk factor for severe symptoms and fatalities from the virus: in England, an estimated 62% of hospital deaths were overweight or obese individuals (Williamson et al. 2020) and diabetes was mentioned in 21% of all COVID-19 related death certificates (21 March to 1 May 2020) (Public Health England 2020). Dietary related NCDs are more prevalent in the UK’s most socially and economically disadvantaged communities, which in turn over-represent people of colour. People from the UK’s most deprived areas are twice as likely to die of COVID-19 (Public Health England 2020). Black and Asian COVID-19 cases occurred at around double the rate they would be if proportionate to their presence in the population (ICNARC 2020) and suffered 10–50% higher risk of death than for white people (Public Health England 2020). Other inequalities that have been exposed include COVID-19 outbreaks in food production settings including meat processing plants (Wales Online 2020) and vegetable packing units (BBC News 2020), drawing attention to low-paid, precarious workers and risky work conditions in current food systems. Such interconnected inequalities demonstrate the need for more just food systems, and show that negative impacts are experienced inequitably when erosion of food system resilience is exacerbated.

### Theoretical framework: Seeking just, people-centred transformations

To understand how changes generated by a pandemic might promote food justice we take a systems perspective to transformation. Food systems are complex and heterogeneous, comprising multiple socio-ecological actors, activities, processes, and factors which interact in often unpredictable ways (Ericksen 2008). Previous transformation of food regimes has occurred through a combination of gradual ongoing change and external shocks to systems (Pereira et al. 2020). The disruption created by COVID-19 opens opportunities for innovation and for less-powerful actors to nudge the regime towards desirable longer-term outcomes: resilient food systems that are sustainable, just and inclusive (Pereira et al. 2020; UN 2020). Justice is too often neglected in food policy discourses, especially at times of crisis (Maughan et al. 2020). The COVID-19 pandemic illuminates multiple systemic injustices connected to food including lack of affordable, good quality and nutritious food for all; poor pay and working conditions for actors within the food system; concentration of power in the hands of a few actors; and limited opportunities for participation in food systems transformation. Investigating these revealed some ways in which food systems need transforming to become more just.

Our research found promising examples of disruption being harnessed to promote transitions founded in principles of justice—just transitions, but more determined orientation towards food justice is required to achieve enduring people-centred transformation. A people-centred approach is central to equitable food system transformations, understanding food justice as “the struggle against racism, exploitation, and oppression taking place within the food system that addresses inequality’s root causes both within and beyond the food chain” (Alkon 2014; Hislop 2014, p. 19). A transformative understanding of food justice recognises that those communities and individuals most marginalised in the food system need to take a central role in determining food system futures (White 2011; Alkon 2018). Food systems based on justice and resilience actively work to address the five faces of oppression (exploitation, marginalisation, powerlessness, cultural imperialism, and violence) within current food systems (Young 1997, p. 151; Slocum 2018). A people-centred approach aims to democratize and redistribute power within food systems by enabling inclusive access to participation in the food system (including the growing, harvesting, gathering, processing, and distribution of food), as well as universal access to sustainable, healthy, culturally appropriate and affordable food (Alkon 2018). The goal of people-centred transformation is challenging unjust power relations at all levels and for all actors (Samuel 2007, p. 617). This hinges upon transformative democratic processes which engage all citizens, as well as undocumented and disenfranchised people (Levkoe and Sheedy 2019, p. 321). We understand people-centred food systems through a framework that includes five core transformational processes (Table 1). We suggest a resilient and just food system can be measured against these five processes for people-centred transformation.

**Table 1:** People-Centred Transformation: A framework of transformational processes

1.	Processes of democratisation hinge upon a rights-based approach to growing, selling and eating sustainable and healthy food
2.	Redistribution of power requires people-centred change within organisations and businesses, and food society more broadly
3.	People-centred approaches strengthen people’s opportunities and abilities to participate meaningfully in food systems
4.	Equity and justice means including engaged citizens, undocumented and disenfranchised people.
5.	Transformative governance, roots food policy in principles of equity and ecological sustainability, with meaningful engagement from civil society, specifically people that produce, harvest, gather, process, distribute and eat food

Based on Samuel (2007), Levkoe and Sheedy (2019), Nicol and Taherzadeh (2020)

## Research design and methods

Our research considered how actors in UK food systems experienced and reacted to the immediate outbreak of the COVID-19 pandemic. While our framing and discussion take a holistic consideration of the food system, our data collection have been necessarily bounded, based on resource constraints that focused our attention on three types of actors across food systems: producers, community organisers and consumers. The research design was shaped by a context of a constantly evolving pandemic, and the urgency of gathering rapid evidence to inform policy responses. To maximise opportunities for immediate data collection we adapted ongoing research projects to include COVID-19. Methods and findings reported here focus on data related to the pandemic rather than to each broader research project. Ethical review and approval of all research practices were granted in compliance with Cardiff University’s ethics policies and procedures (CU 2021). Data collection focused primarily in Wales, allowing detailed investigation of one case study region with good opportunities to learn about globally connected food systems. Wales offers valuable insights to food system vulnerabilities: prior to the pandemic, an estimated one in four people lived in relative poverty (Welsh Government 2019b). Wales is highly reliant on fresh produce grown elsewhere (Welsh Government 2019a). Horticulture accounts for only 0.1% of total agricultural land-use (Welsh Government 2019c) and Welsh land currently provides just 5% of the population’s recommended five-a-day portions of fruit and vegetables (Wheeler 2016) (Table 2).

**Table 2:** Data collection methods

	Producer data	Community activists and organization data	Consumer data
Data collection tool	Surveys Online workshop discussion	Key stakeholder interviews Participant observation in network meetings	Semi-structured interviews
*N*	34 (survey participants) 42 (workshop participants)	4 (interviewees) 34 (meeting participants)	49
Analysis	Descriptive statistics, Qualitative thematic analysis	Qualitative thematic analysis	SPSS descriptive statistics

To understand impacts on fresh food producers we worked with Food Sense Wales (a charity working to influence food policy in Wales), Peas Please (a UK-wide partnership aiming to increase vegetable consumption) and Tyfu Cymru (a project supporting development of Welsh horticultural production) to gather data from fruit and vegetable growers. An online survey was promoted during the first three weeks of lockdown in the UK. Invitations to participate were sent to a database of 150 producers held by Tyfu Cymru which includes all commercial horticultural operations in Wales. Additional participants (e.g., community growing enterprises) were recruited via social media and through organisational networks. Responses were received from 34 growers focused on fruit and vegetable production from around Wales, representing a range of business types and sizes. Tyfu Cymru confirmed that respondents reflected the diversity of producer types found nationally. Survey data were analysed to identify trends in the impacts being experienced and themes in the experiences producers described. These were shared with participants during an online video call with respondents, other growers and interested organisations to reflect on the results, during which we sought to identify consensus on support required from Government. Additional insights were collected through interviews with two Welsh seed producers that supply commercial and home growers.

Insights from community activists and organisations were gathered through four semi-structured interviews with prior contacts known to be active in local crisis responses. Participants included a social enterprise salad producer, a city-level food planner, a horticultural producer, and a community organiser. Discussions led to the convening of an online network to support community organisers by sharing good practice. This forum grew to include over 40 organisers from community food initiatives across Wales and further data were collected by participant observation in eight network meetings in which 34 members were present. Interview data, meeting minutes, and meeting recordings were analysed using qualitative thematic analysis with a framework focusing on immediate impacts of the pandemic and the shared challenges and responses discussed by participants. Several key cases raised by participants are included to illustrate the main themes that emerged.

Consumer experiences were captured through semi-structured interviews with 49 households: 19 had joined a community supported agriculture (CSA) vegetable box scheme that provided a bundle of seasonally available vegetables on a weekly basis in South Wales in 2019 and 30 were randomly selected as a control group to represent those households not participating in a CSA vegetable box scheme. Random selection was done through interview requests of shoppers outside of a major retail supermarket. The household interviews form part of a larger research project, TGRAINS, funded by UK Research and Innovation, looking at how relationships in the food system drive changes in household food culture. The interviews collected data on changes in household food culture, focusing on food purchasing, preparation and consumption. Baseline interviews were conducted with households in summer and autumn 2019. The TGRAINS project originally planned to look at the impact of joining a CSA initiative on household food culture and to compare these changes with the control group, where we anticipated little to no changes. However, the COVID-19 pandemic created a society-wide intervention that led to the research opportunity to understand how households’ food culture changed as a result of the pandemic and lockdowns. Follow-up interviews were conducted in May and June 2020 to look at changes in household food culture. The interviews lasted approximately 45 min and were conducted online or by phone.

## Results

### COVID-19 impacts on food systems

#### Impacts on producers

**Fig. 1:**
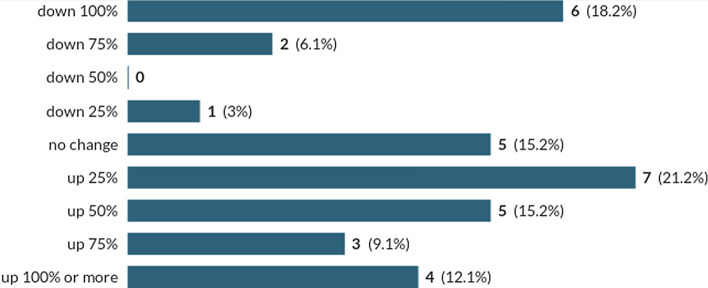
Changes in demand for Welsh growers’ produce during the first month of COVID-19 impacts

Food businesses were among the first to be affected by COVID-19 as lockdowns prompted panic-buying and forced sudden closure of many food outlets. Our survey found that most growers experienced sudden and dramatic increases in demand for fruit and vegetables based on comparison with expected trade for the time of year (Fig. 1). Those who initially experienced dramatic loss of demand tended to be supplying catering trade or selling through local markets or farm shops which ceased trading. Growers generated little waste as they quickly sought alternative sales routes. Several interviewees perceived that accessing new markets and direct sales to consumers was easier in larger urban areas with more affluent populations and with established social networks. Producers without the means to access and distribute to these markets had short-term surpluses which were wasted or sold at reduced prices. Producers were also concerned about the potential lack of security and continuity of new direct sales routes once lockdown restrictions ease and other supplies resume.

Growers reported how the pandemic affected their business with the most common challenges being loss of usual customer base and routes to market and staffing pressures because of absence, illness, and additional tasks. These created an intense workload exacerbated by new administrative demands such as establishing new online sales systems. One grower described the “logistical nightmare” of switching from supplying six restaurants to fifty households:

Catching the orders seems to be quite easy but really labour intensive! So, for every person who orders I might have had like two or three messages or emails with them! And when that’s multiplied by forty, it’s a massive amount—Salad grower

Growers had to adapt to social distancing protocols and safe practices for deliveries, challenges they met rapidly. The largest company recruited 25 additional staff to increase capacity, while smaller owner-operators increased their working hours, negatively impacting wellbeing. Horticulture in Wales employs relatively few seasonal workers so was less affected by labour shortages caused by migration controls (The Guardian 2020). But the growers we surveyed were missing small numbers of workers, which for their teams represented a significant proportion of capacity.

Producers were also concerned about availability of supplies, with seeds a key input affected by the pandemic. Interviews with seed producers highlight vulnerabilities in the food systems to extreme events such as the COVID pandemic, with potentially drastic impacts on production. For example, an estimated 80% of organic vegetable seed is currently imported by the UK. The vulnerability of these supply chains for key inputs is one indication of how resiliency of current food production systems is threatened.

Survey respondents were broadly optimistic about surviving the pandemic and harnessing emerging business opportunities. By responding quickly and flexibly, producers felt they had demonstrated their resilience, and that they were valued by consumers. However, adapting their business operation to maintain supplies to customers had come at considerable financial and work-load cost. Producers expressed frustration that this was under-appreciated by decision-makers, who they felt had failed to invest in the sector or recognise its socio-ecological value. Consensus was that Welsh horticulture could scale-up production if the government would provide small capital grants or loans to invest in site expansion and infrastructure. Several research participants also spoke about barriers in accessing land, particularly for new entrant farmers, representing a missed opportunity to quickly establish new farm businesses and indicating that access to land as a food producer is not equitable. The pandemic therefore highlighted the economic vulnerability of Welsh horticultural producers and their marginality relative to other agricultural businesses and food system actors. Their situation also revealed barriers to increasing production of fresh foods within current food systems, including inaccessibility of farm support schemes and lack of public subsidy, such as those provided under the Common Agricultural Policy, relative to other forms of agriculture.

#### Impacts on consumers and food consumption

ANOVA results show that mean household income (equivalised household disposable income, using the modified Organisation for Economic Co-operation and Development (OECD) scale) for CSA members in our study was higher compared to the mean household income for the randomly selected control group (*F*-value 13.707; *p*-value .000). Mean income in the UK is £35,300 and median income is £29,400 (Office for National Statistics 2020). In our study, CSA members had a mean income of £35,254 (SD £11,269) and a median of £35,714, whereas the control group had a mean income of £28,836 (SD £10,136) and a median of £29,850. Our CSA participants were also on average slightly younger than the people in our control group, and more likely to have dependent children at home. Women interviewees were more common among the CSA members (68% female 32% male) than the control group (58% female, 42% male).

86% of CSA members and 49% of control group members reported changes to their food shopping because of lockdown and the necessity of reducing trips away from home. For many people this meant reducing the variety of places they shopped and going mainly to the closest supermarkets with the largest range of produce. Overall, 52% of CSA members and 37% control group members changed online shopping behaviour. We found that most participants wanted to increase the percentage they bought online, so access to delivery slots was an important factor governing changes in behaviour. Some people in the control group qualified for priority delivery at supermarkets that were participating in government schemes to prioritise delivery to people “clinically extremely vulnerable to coronavirus” (UK Government 2020a, b) and had shifted their shopping to participating local supermarkets. Others would have welcomed similar supermarket delivery slots but were not able to get them. However, while some of those who wanted such access did manage to eventually to find slots and buy more online, others consciously reduced the amount they bought online to allow more access for vulnerable people.

66% of CSA members and 20% of control group members reported seeking information about and sourcing local food. This reflects our findings above, and elsewhere, that interest in local and regional food has substantially increased. A survey of 101 box schemes during the first six weeks of lockdown found veg box sales had doubled with 82% having waiting lists closed to new orders (Wheeler 2020). The two principal reasons given for this change were sourcing food that could not be purchased from supermarkets and supporting local businesses. One interviewee said:

Trying to be helpful to the local community…the one thing that’s really sort of disturbing has come to me, is really about making sure that people keep their jobs. This is the worry I have in my head. I am lucky because I work in an organisation which is required (i.e., NHS); in all this time we haven’t stopped working. So, any sort of way of keeping food people / this industry going. I was impressed by how some of the local businesses adapted so quickly to delivery. If they can do that and provide a sort of healthy version responsibly sourced version, fantastic, if you can get that going. It could be a game changer for a lot of things.

As farmers’ markets closed, we found many could no longer access local produce in their usual way, but some participants found new ways of sourcing food via online or phone delivery/collection services quickly established by local producers. These were usually found via social media and word of mouth.

With most of the public suddenly at home, interest in growing vegetables increased. Three-quarters of control group households interviewed had started growing vegetables for the first time during lockdown. Community growing projects described being flooded with messages from people wanting to grow and share produce. Several innovative campaigns to support and encourage home growing emerged; community organisers made videos, ran webinars, and distributed seeds to support this new enthusiasm. Community food network participants felt the crisis had galvanised public interest for regional food consumption, food growing and thinking about where food comes from. In our household interviews, 66% of CSA members and 40% of control group members were also spending more time cooking. However, they often tended to report eating more, and not necessarily as healthily as they did before lockdown: it was common for participants to say they were consuming more snacks, chocolate, deserts and alcohol, and that they did not view these changes in their diets as desirable in the long term.

Loss of income due to COVID-19 and lockdown has meant that some people are struggling to buy food. 20% of the control group were finding it hard to afford their weekly food purchase or were not sure they could afford it. The role played by voluntary organisations and local authorities in food provision increased for some people in our control group, in addition to those receiving help from friends, family, neighbours and mutual aid groups with food shopping and collecting medication. A common comment made by people in interviews was that they were ‘managing’, and that ‘other people had it worse’, but it seems likely that households’ ability to access enough food to meet nutrition needs has decreased (although participants did not explicitly describe their situations as such) within our control group if we compare participants’ circumstances in 2019 and 2020. This did not arise in the data from CSA members, which likely reflects the higher average incomes of CSA members.

#### Impacts on community action

A notable impact of the crisis has been the rapid mobilisation of community networks and solidarity efforts. Self-defined Mutual Aid and COVID-19 community response groups have sprung up around the UK. In Cardiff, over 20 neighbourhood groups were established, gaining hundreds of members within days. Many now have 1000+ members in their Facebook groups—a primary way of organising. The principal functions of mutual aid groups have been to support neighbours in self-isolation by collecting food shopping and prescriptions and sharing advice and information. They have also become forums for sharing food and plants and have linked with other community efforts coordinated through charities, and community and religious groups focused on food and meal provisioning. Pre-existing community food projects described huge increases in offers of help with food growing, cooking and distribution.

Many local authorities rapidly developed programmes for distributing food packages to those most vulnerable to food poverty and illness. However, research participants noted some officials were referring people to mutual aid groups for support, as happened elsewhere in the UK (Ruiz Cayuela 2020). This places pressure on community solidarity efforts and highlights the inadequacy of pre-existing food poverty responses, with a risk of support suddenly being discontinued. While the community response was overwhelmingly positive and has shown the underlying desire for cooperation, it is not a sustainable response to food insecurity as mutual aid group members face burnout or return to work.

Despite the clear connection between diet and increased morbidity from COVID-19, participants highlighted that local food responses often followed a food bank model of distributing surplus food and vouchers from supermarkets, so lacked fresh, healthy produce. Food banks often will not handle fresh produce due to the additional food hygiene and storage requirements. While many local food businesses and community groups stepped in to fundraise and provide healthier cooked meals or donate food for vulnerable groups and National Health Service staff, the primary response from local authorities and charities was fairly disconnected from regional local food supply. As the city-wide food planner remarked:

The supply chain side of things is one of the things we were looking at, I think it’s always been an area we’ve been slightly weaker on […] it’s not been priority, on the radar, and I think that’s something that will start to become more of a priority

Local producers wanting to donate or redirect sales to the food insecure had no clear route to those in need, while planners wishing to procure from them lacked the necessary distribution systems. This was identified to result from pre-existing fragmentation of the food system and lack of local governance. Small-scale producer networks expressed a desire to be part of food poverty responses and were working on justice-based models of social and community farming aiming to address inequalities. Food banks and the distribution of surplus food was recognised by community organisers as not addressing the root causes of food inequalities, reinforcing an issue now commonly acknowledged (Denning 2019; O’Connell et al. 2019).

### Resilient responses & limits to success

The previous section demonstrates how the shock of a global pandemic has impacted food systems, exacerbating vulnerabilities and threatening resilience, with the most vulnerable producers and consumers being disproportionately affected. We now examine food system actors’ responses, exploring their potential to promote food justice, as well as limiting factors.

#### Resilient supply chains for fresh produce

During the crisis Welsh producers demonstrated their value as suppliers of fresh produce, able to respond quickly and flexibly to market changes to feed people healthy food. Horticultural producers reported changing crop plans and increasing production as well as turning over new land to grow staple vegetables for local markets. There was strong consensus from research participants that Welsh growers are willing and ready to increase production should high levels of demand persist. This is significant given predicted challenges in sourcing fresh produce imports due to COVID-19 impacts on global production (Garnett et al. 2020). Increased domestic horticultural production can support a move to healthier diets, while reducing the ecological impacts of agriculture (Lang and Schoen 2016; Willett et al. 2019). However, participants emphasised that expansion is only possible through increased investment in infrastructure, land, and coordination by national and local governments. Small and medium enterprises lack capital to fund expansion; securing loans is particularly difficult for those operating on rented land. Some operate close to the limits of economic viability and cannot afford to employ additional skilled workers or invest in new equipment or infrastructure. It tends to be bigger businesses that can lever investment, so growth further concentrates with wealthier producers. Most agricultural subsidies are unavailable to those operating on less than 5ha, which excludes smaller, highly productive growers; public subsidies in Europe also typically focus on non-horticultural production. If expansion of horticulture is wholly market driven, agroecological and small- to-medium-scale producers are unlikely to benefit due to financial limits on their capacity. Those interested in establishing new growing enterprises face considerable barriers to entry (Taherzadeh 2019), as do farmers of colour (Levkoe and Offeh-Gyimah 2020). Producers or those who would like to produce food therefore face multiple factors that marginalise them or reduce their power to influence food systems.

One successful initiative supporting new and existing growers is the Planna Fwyd/Plant Food Collective, Machynlleth. Building upon existing programmes and community networks, Planna Fwyd brought together community members, farmers and growers to increase local food resilience at the start of lockdown. They directed people to sourcing seeds and compost; organised sharing of machinery, tools and manure, and volunteering on local farms; obtained land for vegetable production; directed consumers to producers; and distributed seed packs to households. They are now developing a regional crop plan and distribution system.

Our research found other examples of the crisis spurring the development of producer networks, collective distribution structures and increased cooperation. These included farmers reaching out to other farmers to share resources to enable them to scale production. However, it is worth noting that one participant highlighted that crisis is not an ideal time to form partnerships and networks as “when the going gets tough, everyone puts their heads down”. Many producers prioritised meeting immediate demand and lacked time for establishing networks.

Distribution was a key issue highlighted by the pandemic: many areas had under-developed systems, so participants quickly established innovative models linking producers with consumers. The Open Food Network (OFN) is a digital platform cooperative that began in 2012 and supports food enterprises to sell online via food hubs. During the first two months of lockdown OFN experienced an incredible spike in demand as new food enterprises joined and turnover increased sevenfold. Groups of producers also established models like REKO (Hagolani-Albov and Halvorson 2017) where orders are taken through a closed Facebook group and distributed at an agreed local space. However, participants noted that uptake of these systems in Wales remains low due to lack of infrastructure and produce. OFN relies on physical food distribution hubs which require funding and support, and a critical mass of participating local producers. These examples demonstrate potential for technology to support shorter supply chains, and for producer cooperation to enhance food systems resilience by increasing the viability of small-scale producers and reducing their marginalisation as food system actors. However, digital exclusion must be addressed to ensure equality of access to online solutions.

#### Public appreciation of the value of food producers

The pandemic prompted a significant upturn in public appreciation of food producers as key workers vital for national resilience (UK Government 2020a, b). Workers in the food system are among the lowest paid in the UK and can endure poor work conditions (Sustain 2018), a pattern seen across Europe and the minority world. Labour supply is a long-standing vulnerability in UK food systems, exacerbated by post-Brexit immigration policy (Garnett et al. 2020). Horticulture is considered particularly reliant on workers from overseas with the sector arguing that UK citizens do not want these jobs (UK Government 2013). But in response to the challenges of addressing the COVID-19 pandemic, campaigns calling for farm workers received huge numbers of responses from UK citizens, two-thirds of whom had not previously worked in agriculture (Farming UK 2020). The Welsh matching service for land-based jobs had many more candidates—including experienced horticulturalists—than vacancies. The Planna Fwyd Collective coordinated a small ‘land army’ of eager local volunteers to work on farms. These patterns suggest that when employment rates drop UK citizens will consider food production work, particularly if it is promoted as a valuable national service.

However, relatively low numbers of interested UK citizens were appointed or committed to a full season of work. Some new recruits reported unappealing work conditions and poor treatment (Russell 2020). This suggests that unless matched by more appealing conditions and rewards, greater public appreciation of food production work is insufficient to attract people to these careers. This demonstrates that food systems lack capacity to maintain production without access to migrant workers, making them highly vulnerable to crises which affect international mobility. Equitable employment and work conditions in food production should therefore become an explicit goal of sustainability policies (Levkoe and Offeh-Gyimah 2020; Maughan et al. 2020).

#### Community action and local partnerships

Several community growing projects have sprung up in response to the pandemic across Wales. Seed and seedling projects proved popular, sending seeds to households and community groups, coordinating neighbours to share seedlings. A community growing network ‘Edible Cardiff’ sent out hundreds of growing packs to food insecure households through council food parcels and doctors’ surgeries. Community food production was also seen as a promising approach with participants arguing for an increase in communal growing spaces in both urban and rural areas.

Key solutions being developed by participants were local food partnerships and food plans to broker connections between education, health, food and environment, involving local authorities and town councils. In Wales, partnerships active in mobilising effective regional responses to the pandemic included Food Cardiff, Monmouthshire Food Partnership, and Our Food Crickhowell. These agree priorities for the region and guide collaborative action to seek food system change. Local food partnership Food Cardiff for example coordinated immediate responses to food insecurity in the city-region. However, it was felt less suited to rural regions due to a perceived lack of focus on food production and farming communities. Participants also expressed wariness about the sustainability of forming networks under a framework with limited-term funding, suggesting a need for longer-term support mechanisms, such as funding and infrastructure.

Some Welsh regions have taken the opportunity of the lockdown to gather communities for participatory online discussions on developing resilient food systems. As part of a move to promote deliberative democracy, regional food assembly events modelled on people’s assemblies were held in Pembrokeshire, Ceredigion, Powys and Cardiff Capital Region. These brought together actors across the food system, including farmer associations, local councillors, educators, and consumers in mixed-group discussions which exchanged contrasting perspectives on issues. These activities model citizen engagement in public discourse on food systems, a useful basis for more participatory democracy as sought by people-centred approaches to transformation. Those involved are now pushing for regional food assemblies with the aim of affecting policy locally and nationally. It remains to be seen whether they will achieve this, or are offered power to influence democratic processes which are not typically participatory.

## Discussion

### Mechanisms to enable enduring just transformations

Existing transition trajectories bring the danger that crisis further tips the balance of power within the food system, consolidating land ownership and institutional support with large-scale industrial food producers over small-to-medium-scale ecological producers; increasing the market share and control of large-scale retailers over SMEs and regional food distribution networks; and entrenching existing inequities in household food security, access to healthy food, and community resources. As has been illustrated in the previous section, the COVID-19 pandemic offers an opportunity to avoid this path and instead support mechanisms which redistribute power and more strongly embed sustainable and just food practices within food systems. The pandemic has simultaneously highlighted and exacerbated the need for food policy founded in principles of food justice. Five key observations can be derived from our findings:

#### People-centred consumption

Consumers are not always able to exercise choice for just, sustainable foods, as illustrated in the consumer interviews. Access can be limited, and emergency provisioning too rarely includes healthy fresh food. Community-led solutions such as community supported agriculture and food cooperatives present a promising way to enhance consumer equity and justice through meaningful engagement with food systems. Public funding could support community-led action to build enduring support infrastructure and organisations.

#### Enacting and enhancing local democracy

Experiences from Wales indicate a role for local government to work in partnership with producers and suppliers to deliver crisis response which ensures the right to healthy and affordable food is met. They might also lead cross-sector local food partnerships focused on ensuring equitable access to sustainable food. Local food partnerships and assemblies also provide mechanisms for more equitably distributing power to shape food systems through inclusive participation in democratic processes. As outlined in Table 1, people-centred food systems require transformative governance which is rooted in meaningful engagement from civil society.

#### A right to grow sustainable healthy food

The COVID-19 pandemic demonstrates that the UK cannot over-rely on imported food and should consider increasing domestic production of fresh produce, coupled with increasing accessibility. In particular the situation of smaller businesses and agroecological producers needs to be made less marginal so they can expand production and survive crisis. Producers must also ensure just work conditions and rewards for workers. Public sector procurement has the power to exert change by sourcing sustainably produced food that supports public health objectives. But to supply large public contracts, small-scale producers require intermediaries such as processing facilities. Public investment in this infrastructure would enable smaller-scale producers to access new markets and support expansion, creating a more equitable producer landscape. Government could also legislate to redistribute power between primary producers and other food businesses, enabling producers to secure a fair share of profits—a significant step towards more just relationships between food system actors.

#### Redistributing power for resilient supply chains

Improved links between small-scale farms and their communities is a positive outcome of the pandemic. It has bolstered short supply chains able to respond to community needs, demonstrating the importance of adaptive capacities and system resilience. These responses illustrate the need for collaboration and partnership, such as those between local government and producers noted above. In the absence of a formal UK food strategy (which ironically has been delayed by COVID-19), UK Government relied on big retailers, meaning they benefited greatly from increased sales, further consolidating their dominance (Lang et al. 2020). Greater diversity of retail outlets would ensure food producers are not reliant on one route to market and that flows of food continue even when one business type is hit by crisis. Government could support development of alternative routes to market for small scale and medium scale producers and food businesses through business grants or loans, enabling a range of business models to thrive equitably in food systems.

#### Transformative governance

The responses to the COVID-19 pandemic emphasise the need for integrated governance and policy across food systems. Food justice aims to ensure the right to food; we therefore support calls for a national universal food framework to ensure universal access to a healthy sustainable diet, as in Scotland’s Right to Food Bill (Sanderson Bellamy and Marsden 2020; RSA 2019). This would require legislative change and changes in the wider support infrastructure to ensure commitments can be met, whilst aligning with other national services such as health and education. A National Universal Food Framework would re-set norms and change behaviour at local, regional, and national levels. The framework could provide a basis for mechanisms to empower people to engage with food systems and participate in associated decision-making.

## Conclusions

The COVID-19 crisis and lockdown of UK society hugely impacted food systems, with those people already vulnerable most damaged by the effects, reflecting patterns of injustice across the world. Producers, consumers, and community organisations demonstrated considerable commitment and capacity to keep food supplies flowing. However, this has not always ensured that sustainable, healthy food was available for all, or that those who produce food were suitably rewarded or fairly treated. Actors were limited in their capacity to act within the food system, depending on their position and power relative to others because of pre-existing inequalities. Solutions developed as emergency responses used opportunities opened by crisis to shape innovations and demonstrate potential pathways for food system transformation. But in a crisis context, important principles, such as collaboration and participation were de-prioritised, while access to *any food* was prioritised over access to *good food*. Moving beyond this to achieve enduring transformations that embed justice in food systems therefore requires concerted effort to promote people-centred approaches to growing, distributing, selling and eating sustainable, healthy food. Transformative justice requires possibilities for all to participate in food systems, including those individuals and communities currently marginalised. This requires work to dismantle inequitable power relations that exclude some from the benefits of and influence over food systems. It further requires involvement in policy-making processes, including those with lived experience of food injustice. Pandemic experiences demonstrate that equitable, just governance processes and policy frameworks must be in place prior to any crisis to ensure that responses are democratically decided and fair. Since the first COVID-19 lockdown in March 2020, there has been increased efforts to build a bottom-up vision for Wales’ food systems, via People’s Food Assemblies and advocacy by the Food Policy Alliance Cymru (FPAC 2021), as well as other actors, to stimulate the above kind of changes in the Wales Food System. Potential for impact is reflected in debates in Welsh Government over these topics (Welsh Parliament 2020). We are encouraged that these actions represent shifts required to generate a transformation towards resilient and just food systems in the UK.
